# Association of professional pre-qualifications, study success in medical school and the eligibility for becoming a physician: A scoping review

**DOI:** 10.1371/journal.pone.0258941

**Published:** 2021-11-11

**Authors:** Rebecca Erschens, Anne Herrmann-Werner, Tim Fabian Schaffland, Augustin Kelava, David Ambiel, Stephan Zipfel, Teresa Loda

**Affiliations:** 1 Department of Internal Medicine VI, Psychosomatic Medicine and Psychotherapy, University Hospital Tuebingen, Tuebingen, Germany; 2 Competence Center for University Teaching in Medicine, Faculty of Medicine, University of Tuebingen, Tuebingen, Germany; 3 Methods Center, University of Tuebingen, Tuebingen, Germany; 4 Baden-Wuerttemberg Cooperative State University (DHBW), Mannheim, Germany; 5 Faculty of Medicine, Eberhard-Karls University of Tuebingen, Tuebingen, Germany; KTH Royal Institute of Technology, SWEDEN

## Abstract

**Background:**

Literature, individual experiences and common considerations suggest that prior professional qualification can be an advantage for later career development. For instance, in Germany, professional pre-qualification has been honored by medical faculties in selection procedures for several years. However, a systematic evaluation of this relationship lacks. This scoping review summarizes existing literature and addresses the role of prior professional pre-qualifications on objective or subjective study success and the choice of a specialization.

**Methods:**

The scoping review was performed oriented on the PRISMA guidelines. PsycINFO and PubMed databases were searched for relevant studies that included data of medical students with and without professional pre-qualifications. To answer the underlying research questions, this scoping review also includes studies that examine professional pre-qualifications in association with non-cognitive "soft" criteria.

**Results and further directions:**

1055 items were identified and reviewed by two independent reviewers with final 11 studies were included for this scoping review. The results of identified studies that report possible effects of prior pre-professional qualifications are inconclusive but suggest that prior professional qualifications tend not to have rather an advantage on study success. Medical school success for students with prior professional qualifications tended to be below average in the preclinical setting, and there were no differences in the clinical setting compared with students without prior professional qualifications. The influence of professional pre-qualifications has not yet been adequately studied without the moderator variable “waiting time” and “A-levels grade”. The scoping review indicates insufficient number of articles stating a co-relation of prior pre-qualifications and subjective data. Again, the results found are not sufficient to state a clear relationship between professional pre-qualifications and the choice of a specific speciality preference. However, professional pre-qualifications, both in medicine and as "practical experience in rural areas", tend to be beneficial for the choice of becoming a rural physician. Large-scale cross-sectional and longitudinal studies are needed to investigate the influence of professional pre-qualifications on different study trajectory parameters.

## Introduction

The decision to choose a particular subject or profession is a major life decision that decisively determines the further course of an individual’s biography [1]. The study of human medicine is still one of the most popular subjects worldwide. Overall, “doing medicine” has a very similar mechanism throughout the world. In principle, “the human” as a medical “working object” is the same everywhere [2]. However, there are considerable differences with regard to the infrastructure of the medical schools, including selection criteria, as well the content and process of study where one is allowed to become a physician.

### Medicine as a popular field of study worldwide

There are 2,409 medical education institutions worldwide, yet the density is very unevenly distributed. Most medical schools are located in India, with 225, followed by the 153 medical schools in the USA [2]. In the USA, the number of students applying to medical school is increasing, as are the available positions within medical schools [3]. For the 2017/18 academic year, a total of 51,680 students applied to medical school, and 21,338 (41.3%) matriculated (in accordance with Strowd et al., 2019 [3]). In Germany, 98,736 students were enrolled at 29 German universities in 2019 [4]. The requirements for a university place in human medicine are consistently high, and the number of places is often limited. In the German winter semester 2019/20, for example, a total of 41,791 applicants applied for 9,458 places in the human medicine program, i.e. four applicants per place. In the 2019 summer semester, there were 19,704 applicants for 1,678 places, i.e. 12 applicants per place [4].

While the criteria for successful students or physicians are often only roughly outlined, they have a decisive role in the selection of students and the allocation of study places for the subject of medicine. There are descriptions of which competencies should make a "good doctor", such as that of Baumann [5], who defines seven core competencies how to become a good doctor. He describes a doctor as competent if he or she has mastered the process of solving medical problems, has a broad knowledge and clinical skills, has a thorough scientific education, is flexible, knows his or her own limits and continues to develop, and has the right attitude such as empathy and role distance [5]. Furthermore, a successful physician should integrate the competencies of all seven CanMEDS Roles including medical expert, communicator, collaborater, leader, health advocate, scholar and professional. These CanMEDS Roles describe the abilities physician need to have to meet the patients’ health care needs [6].

### Medical education and selection process in Germany

The National Competence-based Learning Objectives Catalog for Medicine (NKLM) was developed on behalf of the Standing Conference of the Ministers of Education and Cultural Affairs of the German Medical Faculty Association (Medizinischer Fakultätentag), the Society for Medical Education (Gesellschaft für Medizinische Ausbildung). The catalog of learning objectives defines competencies that are based on the professional profile of physicians and that should be available upon completion of the respective course of study. In addition to knowledge and skills, these also include higher-level learning objectives such as attitudes, scientific competencies and so-called soft skills [7].

The recent creation of a uniform graduate profile and role definition via the National Competence-Based Learning Objectives Catalogue for Medicine (NKLM) [8] has led to a redefinition of the study objective. In the future, these competencies will also be relevant for the success of medical studies as competence-based examinations play a more relevant role in medical training.

Next to the National Competence-Based Learning Objectives Catalogue for Medicine, the German Master Plan 2020 for medical education provides a graduate profile that, in addition to factual knowledge, is intended to impart skills, abilities and attitudes. Here, the focus is on scientific competence, doctor-patient and interprofessional communication skills as well as a strengthened competence profile for general medicine. In addition to the substantive, methodological and didactic challenges in medical education, the question arises as to what extent selection considerations should occur before students even start their studies, with regard to the later differentiation in the medical specialities.

The Master Plan for Medical Studies 2020 is accompanied by changes in medical studies by focusing on doctor-patient communication and the teaching of scientific skills. Admission to medical studies is to be geared more towards requirements for medical practice, social and communication skills and motivation for medical studies. Selection criteria already in use, such as A-levels, test and interview results or work experience close to medicine, are to be validated, with a focus on predictive validity for social, communicative and scientific competences, as these have been neglected so far.

The previous German system allocated university places according to advance quotas based on the best A-level grades (20%), a waiting period (20%) and individual selection by the universities (60%). The individual selection by the universities (AdH = selection process of university Auswahlverfahren der Hochschulen) is designed to consider other aptitude-related criteria in addition to the A-level grades in the selection of applicants. In particular, non-cognitive factors such as professional pre-qualifications and voluntary service, as well as competitions, were agreed upon. Furthermore, cognitive aptitude tests, such as the Test for Medical Studies (TMS) or psychosocial tests with Multiple Mini Interviews (MMIs) or a Situational Judgement Test (SJT), are still included in the selection procedure [4, 9, 10].

A total of 70% of the study places may be awarded by the higher education institutions according to the results of the higher education institutions’ own selection procedures. The focus can be placed on the following criteria for the allocation of study places: (1) the degree of qualification, (2) weighted individual grades of the qualification, (3) the result of a subject-specific study aptitude test, (4) existing vocational training or work experience, (5) existing special prior education, practical activity or extracurricular achievement/qualification and (6) the result of an interview or other oral procedures.

Especially the pre-qualification in professional practice plays a decisive role in many higher education institutions’ own selection procedures, although the group of students with pre-qualification in professional practice has been studied only little so far.

The issue of professional suitability focusing on professional pre-qualification represents another possibility for student selection. Professional pre-qualification is defined as completed vocational training in healthcare, e.g., nursing,paramedics, geriatric nurse or physician assistant. Until now, the extent to which a selection according to professional pre-qualifications is actually an advantage for future students has been unclear. Often this practice is motivated by the idea that applicants might have better access to the medical content and might also have concrete professional advantages due to their previous experiences. Therefore, applicants with professional pre-qualifications are expected to gather their medical knowledge and perform better in their studies. It is assumed that they can use their professional experience positively in their studies and later in their profession and are better informed than applicants without professional pre-qualifications in terms of what to expect in everyday medical life. In addition, they train and improve their skills through their vocational training. Furthermore, a positive self-selection could result from pre-qualifications, so students with pre-qualifications reflect more on their aims and expectations of their medical study compared to students without pre-qualifications.

### Aim of the study

This scoping review deals with the role of professional pre-qualifications regarding success before and in medical school and speciality choice. To answer the underlying research questions, this scoping review also includes studies that examine professional pre-qualifications in association with non-cognitive "soft" criteria. There are indications from the literature that certain constructs could be considered in predicting study success. For example, specific personality traits such as conscientiousness [11] and the expectation of self-efficacy [12] partially show high correlations with objective study success, as does the learning behaviour of students [13].

Furthermore, several studies showed that vocational interest as non-cognitive “soft” criteria has an impact on study success [14]. In addition to the didactic and methodological challenges of the study program, the question arises as to what extent the selection of students in the run-up to the study program should be considered with regard to the later differentiation of specialists. In particular, according to the current state of knowledge in a few studies from Canada and Australia, the willingness to work as a rural doctor cannot be predicted by selection tests. Rural origin and internships in rural areas during university appear to be the better predictors [15].

The questions should be answered more precisely: Do students with professional pre-qualifications show a more or less successful performance in university? Do prospective students with or without professional pre-qualifications differ with regards to:

- the course of the study, operationalized by objective and cognitive criteria of study success
- subjective data or moderator variables
- their preference for subsequent specialist training.

## Methods

### General methodology and selection criteria

No protocol exits for the review. It was constructed based on the PRISMA Statement [16, 17]. We used to define eligibility criteria to search for relevant literature. The eligibility criteria were as follows (1) *undergraduate medical students* before and in different stages of their university education; (2) items that report data of medical students *with and without professional pre-qualifications*. Professional pre-qualifications are defined as studies or training broadly related to medical education and completed *before* medical school. This includes, for example, training as a paramedic, nurse, or a study of basic natural sciences or health sciences; (3) relevant publications were based on the impact of *professional pre-qualifications* (a) on the studies, (b) with regard to moderator variables, c) on students’ preference for subsequent specialist training. Table 1 gives an overview of our precise operationalization of outcome variables regarding our research questions. (4) Relevant studies can be peer-reviewed articles, systematic reviews and conference papers published in German, French or English in the last 20 years. The search was conducted using the databases of PsycINFO, PubMed, and Google Scholar.

**Table 1:** Overview of precise operationalization on outcome variables.

Outcome Variables		
Research Question	Superordinate	Operationalization/examples
a	Objective/cognitive data in the course of study	Grades; clinical traineeships; preparing a doctoral thesis; repeating exams; dropping out of studies, regular study period; results of selection tests (e.g., TMS, SJTs, MMI, or others)
b	Subjective data or moderator variables	Soft skills; study satisfaction; communication skills, mental health, learning behaviour, study satisfaction, self-organization, personality e.g. conscientiousness, resilience and mental and interprofessionalism
c	Preference for subsequent specialist training	Interest in and initiation of a specific specialist training.

### Data sources and search strategy

PsycINFO and PubMed databases were searched for relevant studies (Title & Abstract screening). Search terms with the following Special Medical Subject Heading (MeSH) terms and combinations were used to search for relevant literature:

((medical student* OR pre-medical student* OR medical subinterns OR medical subintern* OR medical clerk* OR medical intern* OR elective)) AND (pre-preliminary job-qualifications qualification OR “professional qualification” OR “vocational qualification” AND Grades OR clinical traineeships OR “doctoral thesis” OR “dropping out” OR study period OR study satisfaction OR Communication skills OR mental health OR learning behaviour, study satisfaction OR self-organization OR personality OR, team skills OR resilience OR interprofessionalism))

Additional articles were retrieved by cross-checking reference lists of the identified articles. From April 2019 until August 2021, we searched for relevant studies published until August 2021.

### Study selection

Articles were identified using a multi-phase screening process. All articles were checked on the basis of title and abstract and categorized as “eligible” or “ineligible” studies by two independent reviewers. In a next step, studies with the potential to be included were assessed for eligibility on the basis of full-text analyses by both reviewer.

## Results

### General search results and quality assessment

**Fig 1:**
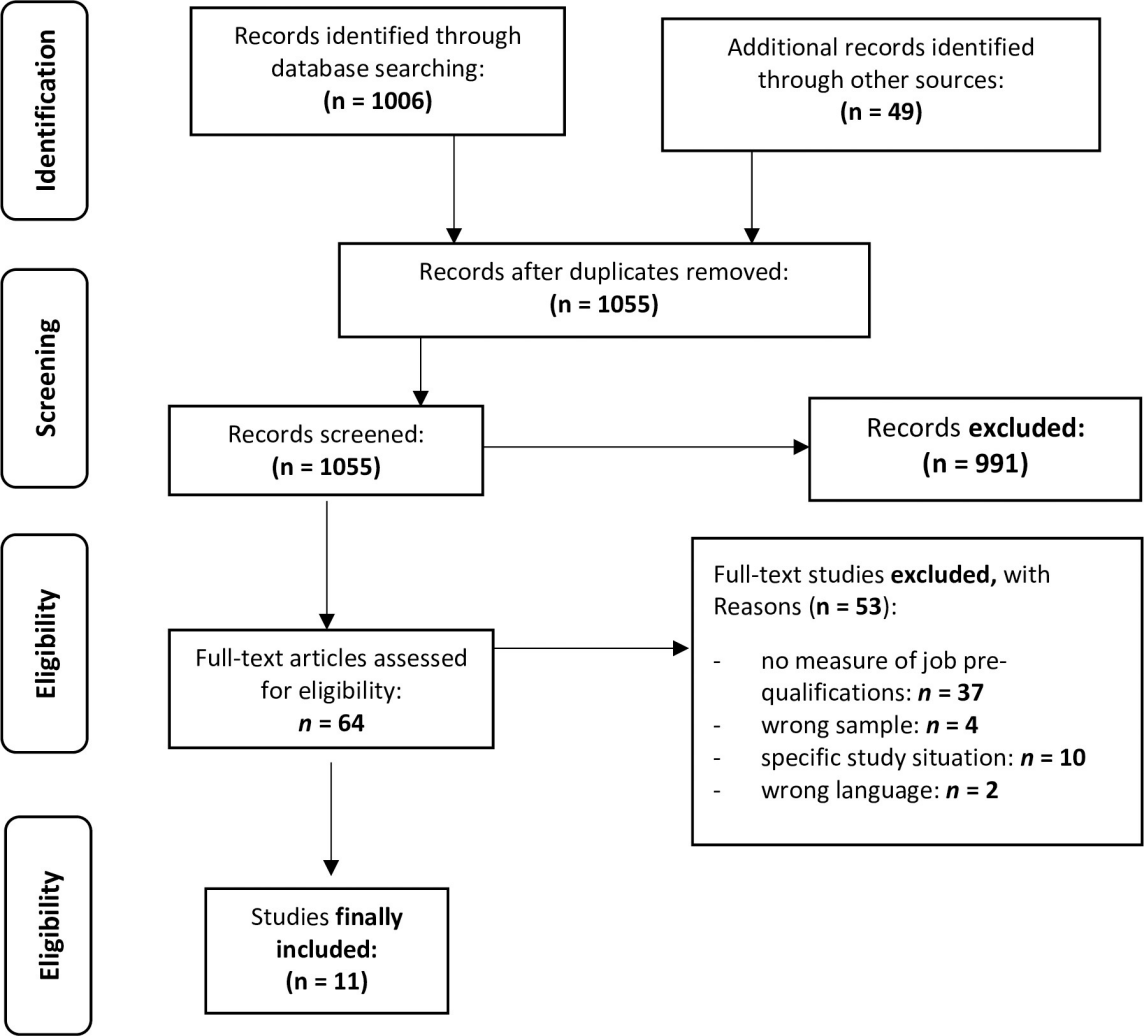
Literature search results in regard to the PRISMA Statement.

In total, 1055 items were identified: 1006 articles were found through databases such as PsycINFO and PubMed and Google Scholar, and the other 49 articles were found through cross- and grey-search strategies that also referenced congress reports and quality reports with results sections. The 1055 articles were reviewed by two independent reviewers based on title and abstract to determine whether an article should be included or excluded; 991 articles were not further included in the full-text analysis. Fig 1 shows a flowchart of the literature search results in regard to the PRISMA Statement adapted to the aims of this scoping review.

There were 64 articles further screened by full-text analysis using PICO criteria, and 53 of the 64 articles were finally excluded. The primary rationale for excluding articles related to the fact that the studies did not examine prior professional qualifications in the strictly defined sense (n = 37), but instead reported preclinical experience or part-time employment in the broad sense, which did not meet the operationalization of the central research question. Of the studies, n = 4 did not report on medical students but, for example, nurses in training. In n = 10 studies, medical students were examined in special study situations to test specific interventions for effectiveness. Here, too, there was confounding of the term "professional pre-qualifications." Two studies were not included because they were not published in English, German or French. Finally, a total of 11 studies were included for this scoping review.

### Responding to the research questions

The following is a summary of the studies identified based on the questions.

#### Association of professional pre-qualifications and objective data

Four of the 11 studies investigated the association of professional pre-qualifications based on objective quantitative data. In the following, we will sub-summarize these studies and highlight the single results. Table 2 gives an overview of the included publications in detail.

**Table 2:** Overview of studies included.

	*Author*	*Article type*	*Sample*	*Frequency of professional pre-qualifications (PPQ)*	*Main outcome*
	*(year*, *country)*		*Characteristics*		
**Research Question a) objective (cognitive) data**					
1	Hampe et al., 2008; Germany	Peer-reviewed	N = 333 medical students;	N = 63 (18.9%^1^) have PPQ	Completed professional pre-qualifications was not correlated with improved academic success.
				N = 51 medical students with medical-associated PPQ were not specified in the analysis	
			n_female_ = 216 (64.9%^1^)		
					Students with professional pre-qualifications passed significantly fewer exams on average (n = 5.8, SD = 6.2) than students without prior vocational qualifications (n = 8.8, SD = 8.7) (t-test: p < .01).
			n_male_ = 117 (35.1%^1^)		
			M_age_ not reported		
2	Heidmann et al., 2017; Germany	Congress paper	N = 277 participants who took part in the university selection process at the European Medical School Oldenburg	Number of students with PPQ not reported	Students with medicine-associated professional pre-qualifications performed significantly better on the overall MMI score (p < .05, r = .14) and on the selection interviews (p < .01, r = .17).
3	Hampe et al., 2009; Germany	Review	Overall number of medical students not reported	Number of students with PPQ not reported	Previous professional pre-qualifications demonstrate no significant difference regarding dropout rates (except for a later start of study at 28–32 years of age).
					The grades for students with professional pre-qualifications were on average slightly worse in the pre-clinical, but not in the clinical, study section than those of the other students
4	Simmenroth-Nayda et al., 2014; Germany	Peer-reviewed	N = 347^3^ participants who took part in the university selection process;	N = 53^3^ (15%^1^) have relevant/health-care associated PPQ	Students with professional pre-qualifications scored significantly better in the selection process of the universities of Göttingen (M = 22.2^4^ points (3.5^4^)/ M = 22.2^5^ points (1.9^5^)) than participants without PPQ M = 19.4^4^ points (3.2^4^)/ M = 20.9^5^ points (2.8^5^)); (δ = 0.74^4^, p < .01^4^/ δ = 0.55^4^, p < .01^5^).
			n_female_ = 247^3^ (71%^1^)		
			n_male_ = 100^3^ (29%^1^)		
			M_age_ = 20.0 years^1^^,^^2^ (R = 17–34 years)		
					The advantage vanishes in the second step of the selection process with A-level grades.
					Participants with professional pre-qualifications tended to score lower in the A-level grades (M = 23.4^4^ points (1.8^4^))/ M = 24.0^5^ points (0.9^5^)) than other participants (M = 27.7^4^ points (1.7^4^))/ M = 26.9^5^ points (1.1^5^) (δ = 2.45^4^, p < .001^4^/ δ = 2.92^5^, p < 0.001^5^).
**Research Question b) non-cognitive data**					
5	Hiemisch at al., 2005; Germany	Peer-reviewed	N = 85^3^ students of medicine and dental medicine at both times;	Number of students with PPQ not reported	Since only 5–6% of the variance could be attributed to the variables age, gender and completed professional pre-qualifications, these variables were not included in the analysis.
			%_female_ = (65%^1^)		
			%_male_ = (35%^1^)		
6	Talwalker et al., 2016; USA	Peer-reviewed	N = 166 students of medical, nursing and physician associate studies;	N = 143 (86.7%) have healthcare-associated PPQ	On the Roles/Responsibility subscale of the Readiness for Interprofessional Learning Scale, students with healthcare-associated professional pre-qualifications scored significantly higher (M = 11.9 points (2.33)) than those participants who had no prior experience (M = 10.7 points (2.19)) (t-test: t(163) = 2.30, p < .05).
			n_female_ = 112 (67.9%)		
			n_male_ = 53 (32.1%)		
			M_age_ = 25.1 years^1^ (2.9^1^)		
7	Paulmann et al., 2016; Switzerland	Congress paper	N = 628 medical graduates 1.5 years after finishing university in 5 cohorts	N = 142 (23%) have PPQ, more than 80% of them healthcare-associated (ca. 19%)	Previous professional qualifications do not influence the dependent variables ("self-assessed medical skills" and "assessment of study satisfaction").
					However, a negative influence due to the delayed start of medical studies could not be shown either.
**Research Question c) preference for subsequent specialist training**					
7	Paulmann et al., 2016; Switzerland	Congress paper	N = 628 medical graduates 1.5 years after finishing university in 5 cohorts	N = 142 (23%) have PPQ, more than 80% of them healthcare-associated (ca. 19%)	Among students with professional pre-qualifications, the field of anaesthesiology appears to be significantly more popular (27%) than among students without professional pre-qualifications (9%)
8	Kesternich et al., 2017; Germany	Peer-reviewed	N = 474 medical students during their clinical period at medical studies	Number of students with PPQ not reported	Students who wanted to become rural physicians tend to have more healthcare-associated professional pre-qualifications, but the difference was not significant (two-sided t-test: p > .05).
				Rractical experience in the health-care sector 53%	
			n_female_ = 299^1^ (63%)		
			n_male_ = 175^1^(37%)		
9	Kopp et al., 2016; Germany	Peer-reviewed	N = 11,462 medical students;	Number of students with PPQ not reported	Healthcare-associated professional pre-qualifications had a modest, nonsignificant, positive influence on the preference toward general practice as residency.
			n_female_ = 6,634 (64%),		
			n_male_ = 3,650 (36%);		
			M_age_ = 25 years^2^		
10	Rourke et al., 2008; Canada	Review	Overall number of medical students not reported	Number of students with PPQ not reported	Students from rural areas are more likely to enter the profession of rural medicine.
					Practical medical work, undergraduate rural training, or postgraduate rural training have a positive influence on entering the profession of rural medicine.
11	Henry et al., 2009; Australia	Review	Overall number of medical students not reported	Number of students with PPQ not reported.	A strong predictor of admissions to rural medical practice is if students resided in a rural area prior to medical school.
					Practical work, especially in the later clinical sections of medical school, also has a positive influence on starting work as a rural physician.

^1^ value self-calculated by author

^2^ standard deviation not reported

^3^summation of values from two different cohorts, self-calculated by author.

^4^ cohort of students in winter semester 2013/14 at the University of Göttingen

^5^cohort of students in summer semester 2014 at the University of Göttingen.

Hampe et al., (2008) investigated the possible correlation of their new-developed HAM-Nat (Hamburger Admission test for Medicine, natural sciences part) and the success of n = 333 (male = 117, female = 216, N = 63 with professional pre-qualifications) students within their first year of medical education [9]. Study success was operationalised on the basis of passed examinations. The authors examined the effects of professional pre-qualifications on success and found that professional pre-qualifications did not correlate with better success. The group of students with professional pre-qualifications passed significantly fewer exams n = 5.8 (6.2) in their first year on average compared to the group of students without professional pre-qualifications n = 8.8 (8.7) (t-test: p<0.001). It is noted that most of these 63 students with vocational training received the university place due to the waiting period quota. However, nine of them were allocated via the AdH. The authors suspect that the greater time lag between the start of their studies and the end of school influenced the number of examinations passed.

Heidmann et al., (2017) examined student candidates who participated in selection procedures at the European Medical School (EMS) Oldenburg from 2012 to the 2016 winter semester (n = 277) [18]. They investigated to what extent cognitive or socio-demographic variables influence the ability to cope with the MMIs. Applicants with completed medicine-related pre-qualifications scored significantly better in the overall assessment of the MMIs (p = .02; r = .14) and the selection interview (p = 0.005; r = .17). The MMIs consisted of a group discussion, a written self-reflection, a patient interview, an interaction exercise, a discussion of an ethical problem, and a selection interview.

Hampe et al., (2009) examined various factors that are assumed to predict study success in medical school [19]. Students with completed vocational training did not differ in dropout rates, except those who had a higher age at the start of university (28–32 years). Mean marks of students with medicine-related pre-qualifications were slightly worse in the preclinical stage but not in the clinical stage.

In a validation study, Simmenroth et al., (2014) examined 347 (male = 100, female = 247) applicants from two cohorts who participated in the AdH of the University of Göttingen in 2014 [20]. In a subgroup, they investigated, among other things, how pre-qualifications affected their performance in the AdH. The test score in the AdH was made up of the points achieved in a structured interview and the MMI and was then offset against the A-level grade. Participants with a completed vocational training tended to perform better on average in both the structured interview and the MMI. On the other hand, the A-level grade was better in the group of participants without work experience, so the slight advantage of participants with professional pre-qualifications disappears in the second step of the calculation of the AdH. The grades of students with pre-qualifications were worse in the preclinical studies but not in the further course of the studies.

#### Association of professional pre-qualifications and non-cognitive data

Three of the eleven studies investigated possible effects of pre-qualifications on subjective, non-cognitive data. Paulmann et al., (2016) report results of a graduate study (1.5 years after completion, in five cohorts, total n = 628) [21]. The authors investigated whether there is an incremental benefit for medical students with pre-qualifications in terms of professional skills, preference for subsequent specialist training and study satisfaction. A total of 62% of the students with training got their places through waiting periods. They found no effect of professional pre-qualifications on self-assessed medical skills and study satisfaction. Also, delayed entry into medical studies does not seem to have a negative effect on study or job satisfaction.

Hiemisch et al., (2005) used a paper-pencil study to investigate various factors that correlate with satisfaction in medical studies [22]. They evaluated 288 students at two different times. Professional pre-qualification was one of the demographic data variables that was not included in the further data analysis due to too little variance explanation.

Talwalkar et al., (2016) examined the readiness of students and trainees in various medical subjects including N = 166 students (male = 53, female = 112) for interprofessional learning [23]. The participants consisted of medical students, nursing students and physician-associated students. Readiness for interprofessional learning was measured using the "Readiness for Interprofessional Learning Scale" (RIPLS). It was found that those participants who had medicine-related professional pre-qualifications achieved significantly higher values on the RIPLS subscale "Roles/Responsibilities" than those participants who had no prior experience. The authors hypothesize that students are likely to have a better understanding of the needs and concerns of different team members based on their own prior healthcare experience. Factors such as the participants’ increased age, previous paid employment in the healthcare sector, or a bachelor’s degree in humanities did not have a moderating effect.

#### Association of professional pre-qualifications and specialist training

Five of the 11 studies investigated possible effects of professional pre-qualifications of medical students’ or physician preference for subsequent specialist training e.g., as a rural physician. Kesternich et al., (2017) address the topic of the shortage of general practitioners (GPs) and rural physicians, which is also considered in the German Master Plan of medical studies 2020 (“Masterplan Medizinstudium 2020”) in the form of an admission quota for prospective rural doctors [24]. This is an additional way to get into medical school as demand far exceeds the number of places available at German universities. The study presents the results of a survey among medical students from Munich (Germany) in the clinical section of their studies. In a multivariate model, the authors determine the effect of socio-demographic factors, typical parameters considered in student selection procedures, and risk aversion on the intention to become a GP or a rural doctor, respectively [24]. Only a) higher self-reported risk aversion, b) lower A-level grade, and c) at least one parent is a medical doctor are significantly and positively correlated with one or both intentions. Professional pre-qualifications in the health sector, the exam score (in Germany: “Physikumsnote”), or the waiting period had no significant effect. They found that medical students who were interested in rural medicine tended to have medicine-related professional pre-qualifications. However, the overall result was not significant.

Regarding the possible association of professional pre-qualifications and medical students’ or physician preference for subsequent specialist training, Paulmann et al., (2016) found a significant popularity of anaesthesia as a continuing education subject among the group of students with completed vocational training (27%) compared to the group of students without training (9%) [21].

Kopp et al., (2016) used an online survey to examine N = 11,462 medical students to investigate factors that influence the choice of specialist training as well as students’ expectations of future careers [25]. For the factor "previous professional experience in the medical field", a weak positive influence on the preference for general practice as a specialist direction was found, but the results were not significant.

In his review, Rourke (2008) highlights various factors that could increase the uptake of rural medicine in Canada [26]. In addition to financial or resource-related aspects, education-related aspects also seem to be a key consideration. Medical students who come from a rural area, have medicine-related professional pre-qualifications or undergraduate rural training, or received postgraduate rural training seem to later practice more often in rural areas.

Henry et al., (2009) also examine the relationship between the selection procedure of medical studies, including previous practical experience and a future career as a rural doctor [27]. Based on interviews with interns who held completed undergraduate medical degrees and interns who completed graduate entry programs, they found a correlation between practical experience in rural areas and a higher interest in a career in rural medicine. According to themthe strongest predictor for specialisation as a rural doctor is having lived in a rural area before studying medicine [27]. Table 2 summarizes the single results from the included articles as an overview in regard to the research questions.

#### Further results

Most of the studies came from Germany with a number of n = 8. The Switzerland and the USA were each represented with two studies. There was one study from Australia and one from Canada. The results showed that Germany had investigated, in the last years whether previous professional experience influenced the success in medical studies. The two studies in Switzerland also presented first steps in this research directions. The different studies from Canada and Australia investigated the preference for rural medicine in particular and were able to show that being from a rural area can be a predictor for the choice of becoming a rural doctor later in life.

## Discussion

This review provides a scoping review of the association of professional pre-qualification on objective, subjective data and preference for specialist training. The training has been honoured by medical faculties in Germany in the selection procedures for several years. This is one of many ways universities in Germany try to narrow down applicants, as demand far exceeds the number of places available at medical faculties. However, delayed graduation and entry into the profession is critically discussed for various reasons. It may be suspect that physicians with previous experience in the medical field have advantages if they pursue a medical career in a field they already know from a non-physician perspective. However, a systematic assessment of this relationship is lacking [28].

### Professional pre-qualifications on objective study criteria

This scoping review addressed the question of how professional pre-qualifications affect different study trajectories and selection parameters. Four studies [9, 18, 19, 29] reported possible effects of prior professional qualifications on objective and cognitive study data. They evaluated the influence of exam grades in preclinical and clinical settings, dropout rates, A-level grades and performance scores on selection interviews such as the MMI. The study situation and a possible effect of prior professional qualifications is inconclusive but leans towards a rather negative impact. The results showed that medical school success for students with prior professional qualifications tended to be below average in the preclinical setting, and there were no differences in the clinical setting compared with students without prior professional qualifications. Students with completed vocational training did not differ in dropout rates, except those entering university at a higher age (28–32 years). Students with professional pre-qualification scored higher on the overall MMI assessment. In contrast, the A-level scores are better in the group of participants without work experience.

Thus, it is discussed that some of the lower performance among students with prior professional qualifications can be attributed to lower A-level grades [9, 19, 28] since the professional activity is often pursued to bridge the waiting period until university admission. In addition, formerly employed students are usually exposed to longer periods of demanding school or academic learning, as well as a potentially changed family situation or increased life demands due to the loss of income. This can result in less time devoted to studying. Formerly employed students may enrich student discussions with other perspectives and increase the heterogeneity of the student body, but this is difficult to quantify [30].

Based on current evidence, A-level grades and aptitude tests are about equally capable of predicting students’ cognitive success and perseverance in the first semesters, but the effect may be lost in the clinical stage [9, 13, 31–33]. In contrast, the dropout rate was related predominately to the age at which students entered the university. Students enrolled through the A-level or AdH quota achieved the best academic performance and lowest dropout rates. The performance level of students admitted through the waiting period quota and the post-delay procedure was lower, and more than 20% of them dropped out during the preclinical study period, half of them due to poor performance. A-level grades and age have different prognostic importance for study performance and persistence rates. Both factors have a negative effect on the prognosis in the group of students who were admitted through the waiting period quota.

In summary, the influence of professional pre-qualifications has not yet been adequately studied without the moderator variable “waiting time” and “A-levels grade”. It would also be interesting to more closely examine students who are admitted through the additional suitability quota (“Eignungsquote”) in order to sharpen a possible influence of the previous professional qualifications. Based on this suitability quota implemented by all medical schools in Germany, applicants are selected independent of their school grades following a 100-point model including professional pre-qualifications [34].

### Professional pre-qualifications on subjective study criteria

In the second research question (research question b), the scoping review examines the effect of professional pre-qualifications on subjective study progress data, such as "soft skills". These include, for example, competence in the interpersonal area, ability to deal with other people or complex skills that enable the individual to take control of reality in communication and interaction situations according to the needs of the participants. Furthermore, professional pre-qualifications can affect inter-professionalism, self-organization, teamwork, mental health and/or study satisfaction.

Only three studies report a possible effect of professional pre-qualifications on the subjective progress of the studies. Paulmann et al., (2016)report that students with and without professional pre-qualifications did not differ with respect to self-assessed medical skills and their own assessment of study satisfaction [21]. Hiemisch et al., (2005) also examined several factors that correlate with various determinants of student satisfaction in medical school in a paper-pencil study. Demographic data included possible professional pre-qualifications, which were not included in further data analysis due to insufficient variance explanation [22]. Talwalkar et al., (2016) examined the readiness of students and trainees in various medical disciplines with professional pre-qualifications [23]. Here, they found that participants with professional pre-qualifications in medical training scored significantly higher on the RIPLS Roles/Responsibilities subscale than those without prior experience. Thus, they concluded that students are likely to have a better understanding of the needs and concerns of different team members due to their own prior healthcare experience.

However, it should be considered that the number of surveys is too small to assess the effect of professional pre-qualifications. For this part of the research question, it is particularly apparent that it is difficult to differentiate the terminology of "early experience". It seems that most studies dealing with the assessment of prior experience on subjective course parameters focus on different programs and courses in medical school [35].

Furthermore, the results showed that Germany and Switzerland compared to other countries had investigated in the last years whether previous professional experience influenced the success in medical studies [21, 24, 25]. This may be due to the fact that practical skills have become more important in medical studies while classical factual knowledge has become secondary.

### Professional pre-qualifications and preference for later speciality training

The third research question (question c) addressed the effect of professional pre-qualifications on a preference for later speciality training. A total of five studies was found. With the exception of one study (Paulmann et al., 2016 [21]) from the field of anaesthesia, four of the included studies reported a possible association of professional pre-qualifications and preference for general practice medicine and, more specifically, for rural medicine [24, 26, 27, 36]. The study by Paulmann et al., (2016) found a positive association between professional pre-qualification and the popularity of anaesthesiology as a residency speciality [21]. Kesternich et al., (2017) suggest that developed and developing countries face imbalances in the distribution of their physician workforce, especially with shortages of general practitioners and rural physicians [24]. Therefore, there is intense scientific discussion analysing specialisations and location decisions of medical students as well as political measures to reduce the shortage of physicians [37].

The literature indicates factors affecting the probability of rural employment include monetary [26] and non-monetary aspects such as one’s choice of work schedule and part-time work patterns [38] as well as the motivation of medical students [39]. With regard to a possible relationship between preference for rural medicine and professional pre-qualifications, Kesternich et al. (2017) found that medical students who were interested in rural medicine tended to have medical-related professional pre-qualifications [24]. However, the overall result failed to reach the significance level. Kopp et al., (2016) also found a weak positive association between previous professional experience in the medical profession and preference for general practice [25].

In his review, Rourke (2008) highlights several factors that could increase the acceptance of rural medicine in Canada [26]. In addition to financial or resource-related aspects, education-related aspects also seem to play an important role. Medical students who come from a rural area and have completed a medical-related pre-qualification or rural undergraduate training, or have received postgraduate rural training, tend to practice in rural areas more frequently. In the review by Henry et al., (2009), the authors found an association between practical experience in rural areas and higher interest in a career as a rural physician. According to the study, the strongest predictor of later specialisation as a rural physician is if students lived in a rural area before medical school [27].

Again, the results found are not sufficient to state a clear relationship between professional pre-qualifications and the choice of a specific speciality preference. However, professional pre-qualifications, both in medicine and as "practical experience in rural areas", tend to be beneficial for the choice of becoming a rural physician.

### Limitations

To the best of our knowledge, this is the first scoping review that specifically addresses the possible relationship of prior professional qualifications to the three questions above. Overall, the number of studies can be considered too small, and the results are too ambiguous to assess the effect of professional pre-qualifications by using a meta-analysis. Most of the studies in the literature overview identified by questions a) and b) were from Germany and therefore may not be universally valid and transferable The fact that most of the study were from Germany might be the results of the issue that the selection criteria to attend medical school in Germany has been a relevant topic for years and should be adapted to focus more on practical competencies and clinical skills. The selection process is different in other countries, where prior professional qualifications may receive less focus when attending medical school.

For instance, In Germany, professional pre-qualification has been honoured by medical faculties in for several years. However, a systematic evaluation of this relationship lacks. This scoping review summarizes existing literature and addresses the role of prior professional pre-qualifications on objective or subjective study success and the choice of a specialization. Furthermore, it need to be considered that all of the studies concerning professional pre-qualifications were conducted in university cities and therefore might not have validity in rural areas. Thus, there was no significance relationship between healthcare-associated professional pre-qualifications and the preference to become rural physicians found.

Therefore, large-scale cross-sectional and longitudinal studies are needed to investigate the influence of professional pre-qualifications on different study trajectory parameters. Ideally, analyses of incremental validity will be conducted by simultaneously surveying a broad predictor space. For the operationalization of study success and subjective study progress parameters, different criteria should be considered simultaneously.

### Implications and further directions

Unfortunately, the number of studies is too small to assess the effect of professional pre-qualifications Studies have shown that A level grades and aptitude tests are suitable for predicting cognitive success in studies and adherence in the first semesters of studies [9, 31–33, 40]. Hampe et al. pointed out that students who had completed vocational training had poorer cognitive success in their studies [9]. International studies have shown that interviews are not very reliably valid, so standardised MMIs are increasingly used to predict social and communicative skills [41, 42]. In addition, written tests such as the Situational Judgement Test (SJT) are used internationally to predict social competence [43]. The discussion and research of selection criteria in human medicine is part of the general scientific, political, educational and philosophical discussion of what makes a good doctor. In this context, the question rises which soft and hard criteria might predict a good doctor. Hampe et al. (2009) discussed the question of who becomes a good doctor by referring to the high school grades and standardized tests to assess the students’ ability that have acceptable validity coefficients with regard to academic and professional success. Concerning students with medical training and professional experience they found that the dropout rate was the same as that of other students [19]. Just and Fischer (2014) found out that students that were admitted by the quota of best graduation (Abitur-Besten) tend to complete their studies with a better exam grade than students in the other quotas [44]. Simultaneously, the results showed that a good doctor should also have some soft criteria like being empathic and patient-oriented and having high level of professional competency [45, 46].

Furthermore, the current studies only focused on selection criteria for undergraduate medical students in order how to successfully complete medical school. Possible criteria for postgraduation was previously neglected. The relationship between student selection and career success should be investigated more closely in future studies. Thus, it should be considered what makes a good doctor and what competences are necessary in physician’s everyday life.

### Conclusion

Research question a) concerned the relationship between prior qualifications and academic success based on objective data. Our results show that professional pre-qualification was not correlated with improved academic success, students even scored slightly worse grades than students without professional pre-qualifications. Nevertheless, students with professional pre-qualifications performed better in the MMI and in the selection interviews. No significant differences regarding the dropout rates were found.

Research question b) concerned the relationship between prior qualifications and academic success based on subjective data. Students with professional pre-qualifications achieved higher scores in the Readiness for Interprofessional Learning Scale but no significant differences in self-assessed medical skills and assessment of study satisfaction could be found.

Research question c) investigated the interest in and initiation of a specific specialist training. The field of anaesthesiology, general practice and rural physicians appears to be more popular among students with professional pre-qualifications. Furthermore, students from rural areas and those who underwent rural training are more likely to enter the profession of rural medicine.

Differentiated conclusions on the possible contribution of prior professional qualifications require studies that consider subjective criteria such as student satisfaction, learning behaviour and self-efficacy in addition to the objective course of studies, such as specific examination results, duration of studies and number of examination repetitions. From the data obtained in this way, conclusions could be drawn as to the extent that students with professional pre-qualifications are slightly less successful in the preclinical setting than students without pre-qualifications. Furthermore, the several studies showed that students with professional pre-qualifications, both in medicine and as "practical experience in rural areas", tend to choose becoming a rural physician more often.

The results showed that students might acquire specific professional pre-qualification when they preferred future speciality training. Thus, students with professional pre-qualifications might be more interested in different or particular specialities in their practical year than students without professional pre-qualifications.
